# A Severe Symptomatic Case of Amyloid-Related Imaging Abnormalities After Donanemab Infusion

**DOI:** 10.7759/cureus.109045

**Published:** 2026-05-17

**Authors:** Nikitha Harikumar, Medha Gupta, Taysir Al Janabi, Karampal K Mand

**Affiliations:** 1 Internal Medicine, Drexel University College of Medicine, Philadelphia, USA; 2 Internal Medicine, WellSpan York Hospital, York, USA

**Keywords:** alzheimer's disease, amyloid-related imaging abnormalities, aria-e, aria-h, donanemab

## Abstract

Amyloid-related imaging abnormalities (ARIA) are known complications of anti-amyloid monoclonal antibody therapy for Alzheimer’s disease. Although many cases are asymptomatic, severe presentations can mimic stroke or toxic encephalopathy. This case highlights a rare presentation of ARIA with myoclonic movements and acute encephalopathy following donanemab infusion. We report a 75-year-old woman with Alzheimer’s disease who developed acute confusion, hallucinations, and involuntary movements one day after her sixth donanemab infusion. Stroke workup was negative. MRI demonstrated findings consistent with both ARIA-E and ARIA-H. She was treated with high-dose intravenous methylprednisolone, followed by an oral prednisone taper, which resulted in gradual neurological improvement. Clinicians must maintain a high suspicion for ARIA in patients receiving anti-amyloid therapy who present with acute neurologic decline. Early MRI evaluation and prompt corticosteroid treatment may improve outcomes.

## Introduction

Anti-amyloid monoclonal antibodies have emerged as disease-modifying therapies for early Alzheimer’s disease. Donanemab, a newer anti-amyloid monoclonal antibody compared with agents such as aducanumab and lecanemab, targets aggregated amyloid plaques to slow disease progression in Alzheimer’s disease [1,2]. In addition to these disease-modifying therapies, commonly used treatments for Alzheimer’s disease include symptomatic agents such as cholinesterase inhibitors (donepezil and rivastigmine) and the NMDA receptor antagonist (memantine) [2]. However, amyloid-related imaging abnormalities (ARIA), including vasogenic edema (ARIA-E) and cerebral microhemorrhage or superficial siderosis (ARIA-H), represent significant adverse effects [1-3]. ARIA occurs when anti-amyloid therapy, such as donanemab, clears amyloid from cerebral vessel walls, leading to transient disruption of the blood-brain barrier with resultant vasogenic edema and/or microhemorrhage [3]. While ARIA is often asymptomatic and detected on surveillance MRI, severe symptomatic cases may present with encephalopathy, seizures, or focal deficits [4,5].

This case is unique due to the presence of profound hyperactive delirium and myoclonic movements in association with combined ARIA-E and ARIA-H following donanemab infusion. It contributes to the literature by highlighting a severe and atypical clinical presentation requiring high-dose corticosteroid therapy.

## Case presentation

A 75-year-old Caucasian woman with Alzheimer’s disease treated with monthly donanemab infusions (17.5 mg/mL solution) presented after being found down at home following an unwitnessed fall. History was obtained from her husband and daughter due to the patient’s altered mental status. She had received her sixth infusion one day prior and reported headache and lightheadedness afterward. She had tolerated prior infusions without any complications. Her medical history included type 2 diabetes mellitus, hypothyroidism, prior pulmonary embolism and deep vein thrombosis with inferior vena cava filter placement, and uterine cancer status post hysterectomy. She was not on anticoagulation at the time of presentation. Home medications included levothyroxine, rosuvastatin, escitalopram, and ezetimibe.

She was last known to be well approximately two hours before being discovered, minimally responsive and disoriented. On arrival in the emergency department, she was confused, intermittently agitated, and not following commands. There were myoclonic movements of both arms, lasting 10-20 seconds and occurring every two minutes. No triggering or relieving factors were reported. Vital signs demonstrated a blood pressure of 154/95 mmHg and oxygen saturation of 89%. Two liters of nasal cannula oxygen was applied with improvement. Neurologic examination revealed that the patient was awake but disoriented. The Glasgow Coma Scale eye subscore was 4, the verbal subscore was 2, and the motor subscore was 5. Pupils were equal, round, and reactive to light. Sensory examination was intact and symmetric. Cranial nerves II-XII examination was grossly intact. At baseline, she is alert and oriented to person, place, and time with some assistance required for activities of daily living.

Laboratory studies revealed a normal blood glucose level, elevated lactate, mild hypokalemia, normal thyroid-stimulating hormone, and elevated creatine kinase. Folate and thiamine levels were within normal limits. Vitamin B12 was elevated. Ammonia level was within normal limits (Table 1). Rapid plasma reagin was nonreactive. Urinalysis did not show evidence of infection. Urine toxicology screen was unremarkable. Arterial blood gas analysis was not pursued as the patient was not in respiratory distress and did not have a history of chronic obstructive pulmonary disease. Lumbar puncture was not pursued because an infectious etiology was low on the differential diagnosis, given that the patient did not have fever, neck stiffness, or leukocytosis on blood work.

**Table 1:** Laboratory findings and their reference values

Test	Results	Reference range
Potassium	3.2	3.5-5.3 mmol/L
Lactate	2.6	0.5-2.0 mmol/L
Thyroid-stimulating hormone	3.59	0.30-5.00 mcIU/mL
Total creatine kinase	424	34-181 IU/L
Ammonia	44	17-60 µmol/L

Stroke protocol imaging, including CT angiography of the head and perfusion imaging, was negative for acute infarction or large vessel occlusion. The neurology service was consulted and recommended a stat brain MRI study and an EEG to rule out subclinical seizures. Additionally, they recommended starting the patient on high-dose methylprednisolone for three days and loading the patient with levetiracetam due to concern for subclinical seizure.

**Figure 1:**
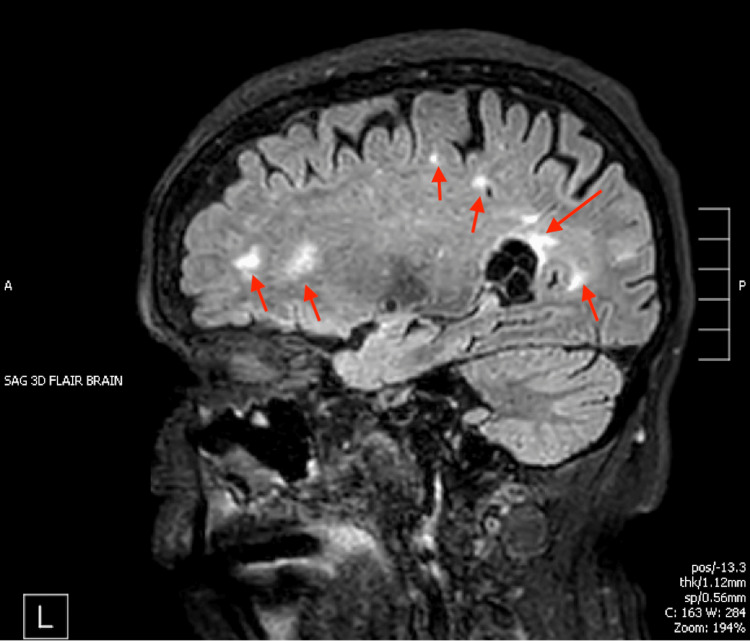
Sagittal FLAIR image demonstrating bilateral subcortical hyperintense signal consistent with vasogenic edema (ARIA-E) ARIA-E, amyloid-related imaging abnormalities with vasogenic edema; FLAIR, fluid-attenuated inversion recovery

**Figure 2:**
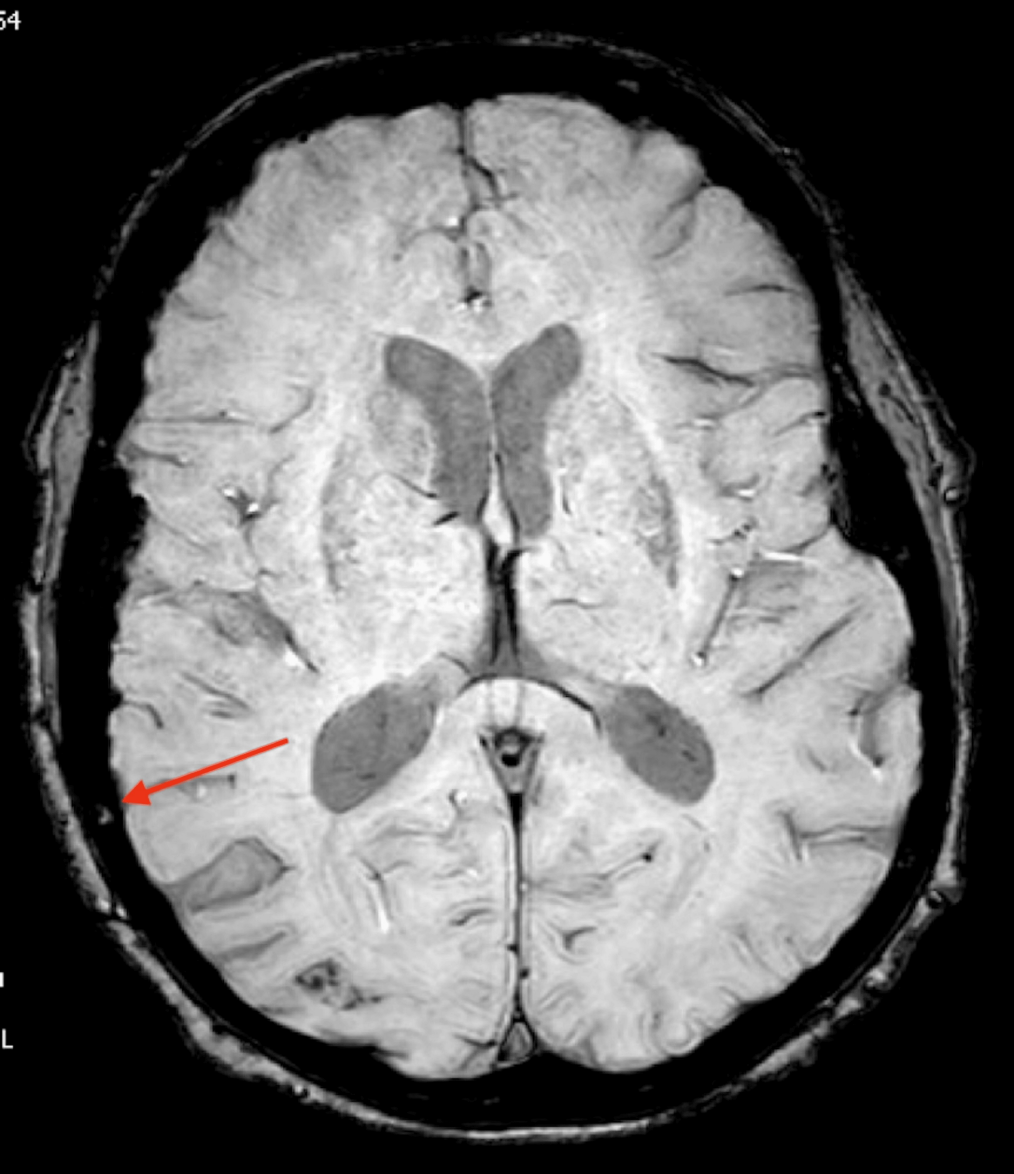
Axial SWI demonstrating a small hypointense focus along the right parieto-occipital cortical surface (arrow), consistent with superficial siderosis (ARIA-H) ARIA-H, amyloid-related imaging abnormalities with cerebral microhemorrhage or superficial siderosis; SWI, susceptibility-weighted imaging

MRI of the brain with and without contrast demonstrated bilateral subcortical vasogenic edema and sulcal effusions consistent with ARIA-E (Figure 1), along with superficial siderosis consistent with ARIA-H (Figure 2). Prior surveillance MRI demonstrated no evidence of ARIA.

EEG showed diffuse slowing without epileptiform discharges, which does not entirely rule out subclinical seizures. Levetiracetam was discontinued. Donanemab was discontinued. Her mental status improved gradually following corticosteroid therapy, and she was discharged on a prednisone taper (35 mg daily for four weeks followed by a decrease of 5 mg weekly) with outpatient neurology follow-up and repeat MRI planned. Outpatient follow-up noted improved alertness, cognition, and neurologic function since hospitalization, with no memory of the acute event.

## Discussion

This is a case of a severe presentation of ARIA in a patient receiving donanemab (Kisunla) infusions for Alzheimer’s disease. Our patient presented with acute encephalopathy and myoclonic movements, as opposed to a more traditional presentation of headache, confusion, or visual disturbances after the first three infusions [1,6].

Donanemab (Kisunla) is an anti-amyloid monoclonal antibody therapy that was approved by the FDA in July 2024 for the treatment of mild dementia in Alzheimer’s disease [1]. Clinical trial data have consistently demonstrated that ARIA is relatively common but often asymptomatic; in the TRAILBLAZER-ALZ 2 trial, donanemab was associated with a substantial incidence of ARIA-E, with most cases occurring early in treatment and resolving spontaneously or with dose modification [1]. These findings are supported by broader systematic reviews and meta-analyses, which confirm a higher incidence of ARIA in treatment groups compared to placebo [2,6]. Despite this, the majority of ARIA cases remain mild and radiographically detected [4].

The clinical spectrum of ARIA is heterogeneous and extends beyond asymptomatic imaging findings. ARIA occurs in about 6% and leads to blood-brain barrier disruption and vasogenic edema (ARIA-E) due to amyloid clearance within cerebral vessels and microhemorrhages or superficial siderosis (ARIA-H) related to microvascular fragility [1,3,6]. Symptomatic ARIA may manifest with headache, confusion, visual disturbances, focal neurologic deficits, or seizures, occasionally mimicking acute cerebrovascular events [7]. Case reports have highlighted presentations resembling stroke syndromes, emphasizing the need for heightened clinical suspicion in patients receiving anti-amyloid therapy [7]. More severe cases, although rare, include extensive cerebral edema, hemorrhagic complications, and even life-threatening outcomes [8]. These observations highlight that ARIA is not merely a radiographic phenomenon but can represent a clinically significant and occasionally severe adverse event.

This patient’s presentation was notable for sudden neurologic decline, myoclonic movements, and fluctuating mental status, initially raising concern for acute ischemic stroke or toxic-metabolic encephalopathy. CT imaging had ruled out acute hemorrhage and large vessel occlusion but demonstrated subtle, scattered bihemispheric subcortical hypodensities, which prompted consideration of ARIA-E and ARIA-H. A subsequent MRI with fluid-attenuated inversion recovery and susceptibility-weighted imaging was critical in distinguishing ARIA from traumatic hemorrhage or posterior reversible encephalopathy syndrome and in establishing the diagnosis.

The case suggests that combined ARIA-E and ARIA-H may manifest with severe movement disorder and fluctuating mental status. Early recognition of this presentation in patients receiving donanemab can allow for prompt discontinuation of therapy and initiation of high-dose corticosteroids, which may facilitate gradual neurologic improvement and functional recovery. As anti-amyloid therapies become more widely implemented in the treatment of Alzheimer’s disease, clinicians in inpatient settings should also consider ARIA in patients presenting with stroke-like symptoms or unexplained acute neurologic decline, as demonstrated in this case. Failure to do so may result in inappropriate initiation of antiplatelet drugs due to suspicion for ischemic stroke and possibly worsen outcomes.

## Conclusions

This case demonstrates that severe ARIA following donanemab may present with acute encephalopathy and myoclonic movements, which can mimic stroke. Early recognition, MRI confirmation, discontinuation of therapy, and high-dose corticosteroids are essential for neurological recovery. Clinicians should maintain vigilance for ARIA in patients receiving anti-amyloid monoclonal antibodies who develop sudden neurologic decline.
